# Prioritization of *Trypanosoma brucei* editosome protein interactions interfaces at residue resolution through proteome-scale network analysis

**DOI:** 10.1186/s12860-024-00499-4

**Published:** 2024-01-26

**Authors:** Naghmeh Poorinmohammad, Reza Salavati

**Affiliations:** 1 Institute of Parasitology, McGill University, Ste. Anne de Bellevue, Montreal, Quebec H9X 3V9 Canada; 2 Department of Biochemistry, McGill University, Montreal, Quebec H3G 1Y6 Canada

**Keywords:** Trypanosomatids, RNA editing, Drug discovery, Protein-protein interaction

## Abstract

**Background:**

*Trypanosoma brucei* is the causative agent for trypanosomiasis in humans and livestock, which presents a growing challenge due to drug resistance. While identifying novel drug targets is vital, the process is delayed due to a lack of functional information on many of the pathogen’s proteins. Accordingly, this paper presents a computational framework for prioritizing drug targets within the editosome, a vital molecular machinery responsible for mitochondrial RNA processing in *T. brucei*. Importantly, this framework may eliminate the need for prior gene or protein characterization, potentially accelerating drug discovery efforts.

**Results:**

By integrating protein-protein interaction (PPI) network analysis, PPI structural modeling, and residue interaction network (RIN) analysis, we quantitatively ranked and identified top hub editosome proteins, their key interaction interfaces, and hotspot residues. Our findings were cross-validated and further prioritized by incorporating them into gene set analysis and differential expression analysis of existing quantitative proteomics data across various life stages of *T. brucei*. In doing so, we highlighted PPIs such as KREL2-KREPA1, RESC2-RESC1, RESC12A-RESC13, and RESC10-RESC6 as top candidates for further investigation. This includes examining their interfaces and hotspot residues, which could guide drug candidate selection and functional studies.

**Conclusion:**

RNA editing offers promise for target-based drug discovery, particularly with proteins and interfaces that play central roles in the pathogen’s life cycle. This study introduces an integrative drug target identification workflow combining information from the PPI network, PPI 3D structure, and reside-level information of their interface which can be applicable to diverse pathogens. In the case of *T. brucei*, via this pipeline, the present study suggested potential drug targets with residue-resolution from RNA editing machinery. However, experimental validation is needed to fully realize its potential in advancing urgently needed antiparasitic drug development.

**Supplementary Information:**

The online version contains supplementary material available at 10.1186/s12860-024-00499-4.

## Background

*Trypanosoma brucei*, a protozoan pathogen, is the causative agent of African trypanosomiasis, endangering millions of human lives and livestock in sub-Saharan Africa [1]. As a prominent member of kinetoplastids, *T. brucei* harbors a mitochondrial genome known as kinetoplast DNA (kDNA). Within this genome, most genes exist as cryptogenes whose transcripts require post-transcriptional RNA editing to gain protein-coding capability [2]. RNA editing is thus an essential facet of gene expression in these organisms. Central to this process is the editosome, a multiprotein complex that orchestrates the Uridylate (U) insertion/deletion editing process, ultimately generating translatable transcripts [3].

According to current knowledge, RNA editing in kinetoplastids involves a coordinated interplay of over 70 catalytic and non-catalytic proteins. These include diverse enzymes, RNA-binding proteins, and other factors whose exact functions largely remain unknown [4]. The holo-editosome (editosome holoenzyme) comprises approximately 40 proteins, distributed across three distinct multiprotein complexes: the RNA Editing Catalytic Complex (RECC), RNA Editing Helicase 2 Complex (REH2C) and the RNA Editing Substrate Binding Complex (RESC) [4, 5]. RECC and REH2C are mainly catalytic; RECC is responsible for the endonucleolytic cleavage, U-indel (insertion/deletion) editing, and ligation of the edited segments. REH2C enhances the accessibility of the editing sites, allowing the other components of the editosome to perform their functions effectively. Meanwhile, the heterogeneous RESC provides the structural framework for the interaction between the guide RNAs (gRNAs) and the target mRNA sequences [4].

The intricate nature of the editosome has made understanding its interactions and underlying mechanism a challenging endeavor [6, 7]. Considering the potential for identifying drug targets by exploring editosome PPIs in *T. brucei*, integrating computational approaches has become increasingly important. Characterizing the PPI interfaces within the pathogen is a crucial for identifying drug targets and designing potential drugs to disrupt these interactions, as seen with the editosome.

Apart from the expensive and time-consuming experimental procedures to identify PPI interfaces, the recent introduction of AlphaFold-Multimer has been a great help in achieving accurate structural resolution of protein interactions [8]. However, pinpointing the most important PPI interfaces and finding the hotspots within those interfaces require further residue-level analysis. To address this, the residue interaction network (RIN) analysis provides a systems-level strategy [9, 10]. This approach identifies central nodes, or hub residues, within the network through centrality analysis of the RIN. Combined with AlphaFold-Multimer findings, this allows for the identification of interaction hotspots.

Moreover, functional annotation gaps are common in Trypanosoma proteins, particularly within the editosome, which is distinct across various life domains. An efficient computational framework could offer a way to prioritize drug targets, bypassing the necessity of gene or protein characterization. This could address a significant challenge in drug discovery. Accordingly, this study proposes a systems-level approach implementing primarily the experimental PPI data of *T. brucei* to reconstruct an editosome PPI network. This serves as the basis for identifying critical PPIs. Residue-level analysis through RINs of each PPI structure helps in recognizing interface hotspots and quantitatively assessing the most important interfaces. Integrating these findings with existing proteomics data from *T. brucei* developmental differentiation, enabled the final prioritization of predicted PPI interfaces within the editosome PPI network.

## Methods

### Editosome PPI network reconstruction

Throughout the study, a set of seventy-four proteins associated with mitochondrial RNA processing in Trypanosoma were employed, data of which can be found in [4]. Identifiers for these proteins were used to retrieve information on their respective interactions from TrypsNetDB for *T. brucei* TREU927. TrypsNetDB is a comprehensive database that houses experimentally substantiated interactome data specific to trypanosomatid proteins [11]. To ensure comprehensive coverage, an extensive literature review was conducted to incorporate the most recent experimental interactions into the final dataset. We also considered the experimental techniques used to validate each interaction, such as affinity purification, immunoprecipitation, and Y2H (yeast two-hybrid). Furthermore, we assigned an assay score on a scale from 0 to 1 to each interaction, reflecting the level of support provided by the number of experimental techniques confirming its validity.

Final interaction data were visualized using Cytoscape 3.9.1. CytoNCA plugin was implemented for the topological analysis of the network [12]. Calculated centrality measures include degree, betweenness and closeness. These three centralities are known to best represent the hub nodes in biological networks [13–15]. To consider the level of confidence for an interaction, assay scores were assigned as weights to the network edges during the centrality analysis.

Many existing studies focus solely on one specific centrality measure, such as degree, which can lead to the loss of valuable information. This study employs a multi-criteria approach, using three centrality measures to identify hub proteins within the editosome PPI network. Multi-criteria decision analysis (MCDA) [16] was used to rank the identified proteins, with equal weightage given to the three centralities. The resulting MCDA score, ranging from 0 to 1 for each protein, signifies its performance across multiple centralities. A higher MCDA score indicates a strong potential for a protein to be a hub, effectively consolidating multiple centrality information into a unified ranking measure. Here, editosome proteins with MCDA score of more than 0.7 were considered as hubs.

### Structure prediction of hub PPIs

All interactors connected to each selected hub protein, supported by at least one RNase positive experimental technique, were fetched from the editosome PPI network. This filtration step aimed to eliminate interactions that rely solely on the presence of RNA, thereby improving the accuracy of PPI modeling and subsequent analyses.

Each binary PPI involving the hub proteins was modeled by AlphaFold_2.0_multimer.v3 as implemented in the Cosmic2 gateway [16]. AMBER molecular dynamics simulation was also configured to optimize the amino acid side chain position of the top-ranked model.

PPI structures were visualized and further analyzed using UCSF ChimeraX version: 1.5 [16]. Residues from the interacting proteins located within a proximity of ≤ 5 Angstroms (Å) were selected and designated as primary interface residues. Predicted alignment errors (PAE) is a quantitative assessment metric from AlphaFold that provides a distance error for every pair of residues. It represents the tool’s position error estimate at residue x when the predicted and true structures are aligned on residue y, with value ranging from 0 to 35 Å. In PPI modeling, low PAE values for residue pairs (x, y) from each interacting protein suggest that AlphaFold has accurately predicted their relative positions and orientations [16]. Thus, primary interface residues with PAEs of ≤ 10 Å were selected and considered as confirmed interface residues used for further analysis.

### RIN analysis

While PAE provides residue-level evaluation of modeled interfaces, it does not inherently capture the biological characteristics of the hub protein. To achieve a holistic understanding of each hub protein’s interactions at the residue level, we reconstructed a residue interaction network (RIN). RINs serve as graphical representation of protein structures, where residues act as nodes and their physicochemical interactions form the network edges.

Using RING 3.0 [10], we generated RINs based on the bound-state structure of the hub protein and its interaction partners. We also incorporated the energy of interaction between residues as weights for the RIN edges.

We performed a centrality analysis on the reconstructed, weighted RINs, following the conditions outlined in the previous section. This analysis identified central residues within the protein; if they coincided with the confirmed interface identified earlier, they were categorized as hotspot residues. We ranked the identified hotspot residues using a dual-criteria approach that considered their RIN-based MCDA score (cutoff = 0.5) and their PAE values.

### Proteomics data analysis

We used quantitative proteomics data from different developmental stages (timepoints) of *T. brucei* TREU927, as generated by Dejung et al. [17]. Missing values in protein abundances were imputed using missForest R package [18, 19] based on the log2-transformed Label-Free Quantification (LFQ) intensities of each timepoint. We performed differential expression analysis (DEA) using DEqMS, a tool that extends the capabilities of the Limma R package [20]. This adaptation involved modifying Limma’s variance prior estimation to account for the relationship between variance and the number of detected peptides for each protein. This modification results in a more accurate, data-dependent estimation of protein variance [20]. We used the resulting fold changes and adjusted *p-*values from the DEA as input for gene/protein set analysis (GSA), facilitated by the “piano” package [20]. Protein sets were assembled based on a gene ontology (GO) term dataset derived from the detected proteome of *T. brucei*, using various sources including AmiGO [21], Blast2GO [22], TriTrypDB [23], Pannzer [24], and UniProt [25]. GSA and DEA were also applied to the editosome subset of the proteomics data where needed.

## Results and discussion

### Editosome PPI network of *T. brucei*

#### Reconstruction of the editosome PPI network

The reconstructed PPI network of editoproteome in *T. brucei* resulted in 90 nodes with 677 edges (Additional file 1). While nodes in the network represent proteins, the edges signify experimentally verified interactions. These interactions were identified using various techniques, including but not limited to Y2H, immunoprecipitation, affinity purification, and fractionation [11]. It is important to note that a single protein interaction may be supported by multiple techniques and could, therefore, have multiple edges in the network. This concept was used as the basis for a scoring system to assign weights to the edges. Consequently, interactions verified by multiple studies received higher scores for subsequent network analyses.

#### Hubs in the editosome PPI network

Hubs were identified and ranked based on Multi-Criteria Decision Analysis (MCDA) of betweenness, closeness and degree centralities (Additional file 2). The top 20 hubs predominantly include RESC proteins, comprising 85% of all known RESC proteins. The predominance of RESC proteins as hubs underscores their pivotal role in Trypanosoma’s RNA editing machinery. One reason is that these proteins often act as the scaffold for editing; thus identification of RESC complex proteins as hubs aligns with their fundamental roles in the RNA editing [3, 26, 27].

For further analysis, 10 proteins were selected as top-rank hub proteins with MCDA ≥ 0.6. As illustrated in Table 1, the majority of these top-ranked hub nodes are also RESC proteins. From all known RESC proteins in the editosome (n = ~ 20), six were identified as top hubs in the editosome PPI network. The remaining top-ranked hubs encompass two editosome ligases, KREL1 and KREL2, along with the REH2C complex helicase and zinc-finger proteins, KREH2 and KH2F1. The two ligases are paralogs and KREL1 is reported to be essential ligase for parasite survival [28]. KH2F1 serves as an adaptor linking the KREH2 helicase with the RESC [4]. A finding also supported by the study from Kumar et *al.*, which demonstrated the critical roles of KREH2 and H2F1 in forming stable mRNA-gRNA hybrid substrates within molecular scaffolds for the editing process [29]. Thus, the centrality of these proteins aligns well with their essential functions in RNA editing.

**Table 1:** List of top ten hub proteins of editosome PPI network in *T. brucei* and their centrality measures

Rank	TriTryp ID	Name	Betweenness*	Closeness*	Degree*	MCDA score
1	Tb927.1.3030	KREL2	1532.5881	0.1580	11	0.8261
2	Tb927.11.16860	RESC3	422.2286	0.1915	16.5	0.7530
3	Tb927.6.1680	KH2F1	1334.1823	0.1712	8	0.7449
4	Tb927.7.800	RESC10	464.2319	0.1903	14.125	0.7122
5	Tb927.9.4360	KREL1	733.3652	0.1733	11.375	0.6860
6	Tb927.5.3010	RESC6	243.2760	0.1891	15.25	0.6847
7	Tb927.10.10830	RESC13	275.5744	0.1881	14.875	0.6825
8	Tb927.2.3800	RESC2	347.8233	0.1947	13.375	0.6792
9	Tb927.8.8170	RESC12a	302.1332	0.1809	14.125	0.6608
10	Tb927.4.1500	KREH2	140.5734	0.1899	13	0.6183

* Degree: Number of direct interactions a protein has; Closeness: Measures how quickly a protein can interact with all others; Betweenness: Identifies proteins critical for connecting others in the network

### Structure-based analysis of central editosome PPIs

#### Modeling hub PPIs using AlphaFold-Multimer

As evident in Table 1, each top-rank hub interacts with multiple partners, denoted by a minimum weighted-degree of 8. We restricted our modeling to PPIs that met with the following criteria: (a) supported by at least one experimental assay performed in the presence of RNase, and (b) highly ranked based on their edge scores, signifying that interactions supported by multiple techniques are considered more reliable.

Twenty-two hub PPIs were ultimately identified, and their 3D interaction structures were modeled using AlphaFold-Multimer (Table 2). Well-known interaction such as KREL2-KREPA1 [3, 30], KREH2-KH2F1 [29, 31], and RESC2-RESC1 [32] ranked among the top PPIs. Moreover, given that the editosome PPI network is highly interconnected, and according to Table 2, the fact that multiple target proteins serve as hubs themselves make these interactions particularly interesting for further analysis.

**Table 2:** Central editosome PPIs in *T. brucei*

PPI No.	Hub		Target	
	Name	TriTryp ID	Name*	TriTryp ID
1	KREL2	Tb927.1.3030	*RESC3*	Tb927.11.16860
2			KREPA1	Tb927.2.2470
3	RESC3	Tb927.11.16860	*RESC2*	Tb927.2.3800
4			RESC5	Tb927.10.11870
5			*RESC6*	Tb927.5.3010
6	KH2F1	Tb927.6.1680	*RESC3*	Tb927.11.16860
7			RESC2	Tb927.2.3800
8	RESC10	Tb927.7.800	RESC3	Tb927.11.16860
9			*RESC13*	Tb927.10.10830
10			RESC4	Tb11.02.5390b
11			*RESC6*	Tb927.5.3010
12	KREL1	Tb927.9.4360	KREPA2	Tb927.10.8210
13			KREH1	Tb927.11.8870
14	RESC6	Tb927.5.3010	*RESC13*	Tb927.10.10830
15			RESC12	Tb927.4.4160
16			*KH2F1*	Tb927.6.1680
17			RESC5	Tb927.10.11870
18	RESC13	Tb927.10.10830	RESC9	Tb927.2.1860
19	RESC2	Tb927.2.3800	RESC1	Tb927.7.2570
20	RESC12A	Tb927.8.8170	*RESC13*	Tb927.10.10830
21	KREH2	Tb927.4.1500	*RESC6*	Tb927.5.3010
22			*KH2F1*	Tb927.6.1680

* The names of target proteins identified as hubs themselves are formatted in italics

The structures of 20 central PPIs were successfully modeled. However, jobs involving PPIs that included the 2167-aa KREH2 ran out of memory due to the current limitations on the total number of residues [8].

Subsequently, PPI models were filtered based on high average PAE (> 10 Å) at their interface, defined as < 5 Å between residues from the two interacting proteins. Models with high PAE values, which imply ambiguity in the interaction details, were discarded [33]. This led to a final set of six hub PPIs with very low average PAEs at their interfaces.

Most editosome proteins and interactions are evolutionary unique and lack sufficient sequence homology for straightforward prediction using AlphaFold2, which relies on multiple sequence alignment (MSA). However, as efforts to improve AlphaFold2’s performance continue, the possibility of accurately studying all hub PPIs in the future remains viable.

Finally, the six accurately modeled interactions included KREL2-KREPA1, RESC2-RESC1, RESC10-RESC6, KREL1-KREPA2, RESC12A-RESC13 and RESC3-RESC2.

#### Interface hotspots identified through RIN analysis

The analysis of complex biological systems in the form of networks can efficiently aid in characterizing the whole system and its individual components [34]. Protein structures serve as an excellent example of such complex systems and can be represented as graphs, where amino acid residues act as the nodes and their interactions as edges. Calculated topological parameters from RINs have shown to correlate with various aspects of protein structure and function. This approach has been successfully used to identify key residues involved in protein stability [35], folding [36], functional and regulatory roles (e.g., active site residues) [37] as well as finding cancer mutation hotspots [38]. More importantly, RINs provide a global perspective on PPI interfaces, mainly by finding hotspots useful for drug discovery and design [9]. Furthermore, since certain critical residues located within the PPI interfaces exert larger energetic impacts on the affinity and stability of the interactions, uncovering these PPI hotspots holds immense potential for not only modulating the PPIs in drug discovery studies but also guiding the design of site-directed mutagenesis experiments aimed at characterizing protein function and interactions. Therefore, to study hub PPIs, we reconstructed RIN of each hub protein in its bound state with its top interactor. Subsequently, centrality analysis was undertaken, focusing primarily on two objectives: (1) Prioritizing the interfaces detected from previous steps; (2) Finding hotspot residues in each interface.

Through RIN topological network analysis, centrality measures were assigned to each residue in the form of a MCDA score. This score was based on betweenness, closeness and degree centrality for each hub. By merging these residue-level measures with PAE values, it became possible to both quantitatively rank, and filter identified interface residues and to detect hotspot residues. Comprehensive results are available in Additional file 3, and a summary of this approach’s general outcome is provided in Table 3.

**Table 3:** Prioritized hub editosome PPIs properties

Hub PPI	Interface length^*^	Average PAE	Average RIN MCDA	Rank ^†^	Top 3 interface hotspots
KREL1-KREPA2	7	4.12	0.70	3	N429, E433, L425
RESC3-RESC2	8	7.40	0.53	6	Y446, H407, L475
RESC10-RESC6	13	2.91	0.58	4	K303, V100, P99
KREL2-KREPA1	8	1.26	0.69	1	F393, Y390, R396
RESC2-RESC1	15	2.06	0.60	2	H345, Y250, R220
RESC12A-RESC13	8	2.56	0.54	4	E644, Y598, R641

^*^ Each residue from the hub protein within the interface may interact with more than one residue from the target protein

^†^ Using dual-criteria ranking approach based on the average PAE and average RIN MCDA

Using quantitative measures and cutoffs, multi-step prioritization was performed on the editosome PPIs to find the most critical PPIs, interfaces, and hotspots, which could significantly help in future drug discovery efforts. These results can also inform functional understanding through experimental mutation assays.

According to our findings, hotspots are mostly enriched with tyrosine and arginine residues. Tyrosine, an aromatic amino acid, often engages in pi-pi stacking interactions with other aromatic residues, significantly contributing to the stability of protein-protein complexes. Arginine, on the other hand, may form multiple hydrogen bonds both within the protein and with the interacting partner. These amino acids have been frequently assigned as interface hotspots in different studies [39, 40].

##### PPI interfaces of central editosome ligases

The editosome ligases, KREL1 and KREL2 share significant sequence homology. Similarly, their binding partners, KREPA2 and KREPA1, also display sequence conservation in specific regions, suggesting functional analogies between the two PPI. Despite these similarities, our findings reveal distinct differences between the two interactions, in which the KREL2-KREPA1 interaction ranking higher and featuring different types of hotspot residues in the identified interface.

Table 1 highlights that KREL2’s central position on the editosome interactome, having a notably higher betweenness centrality compared to its paralog, KREL1. This coupled with its enhanced interface importance ranking, suggests that KREL2 plays a more pivotal role in RNA editing than previously thought. Surprisingly, this contradicts prior studies using knockdown assays, which suggested that KREL1 is essential for RNA editing, while KREL2 appears to have little impact [28]. A widely-accepted theory posits that KREL1 can compensate for KREL2 loss, but not vice versa [41]. Yet, the lack of studies on KREL2 null mutants leaves room for alternative explanation. Our results propose that even a minimal level of KREL2 may suffice for RNA editing, which could explain the lack of severe impact upon its knockdown. Furthermore, morphological changes observed in KREL2 knockdown experiments [41] indicate its importance in the parasite’s biology, potentially during specific developmental stages.

While the KREL2-KREPA1 PPI ranks highest in terms of the structural and network-based metrics used in this study, its potential as a drug target may be compromised by reports suggesting that KREL1 could compensate for the loss of KREL2. However, this compensation mechanism is not yet fully experimentally verified and requires further investigation. If proven otherwise, the KREL2-KREPA1 interaction could serve as a valuable drug target for combatting *T. brucei*.

**Fig. 1:**
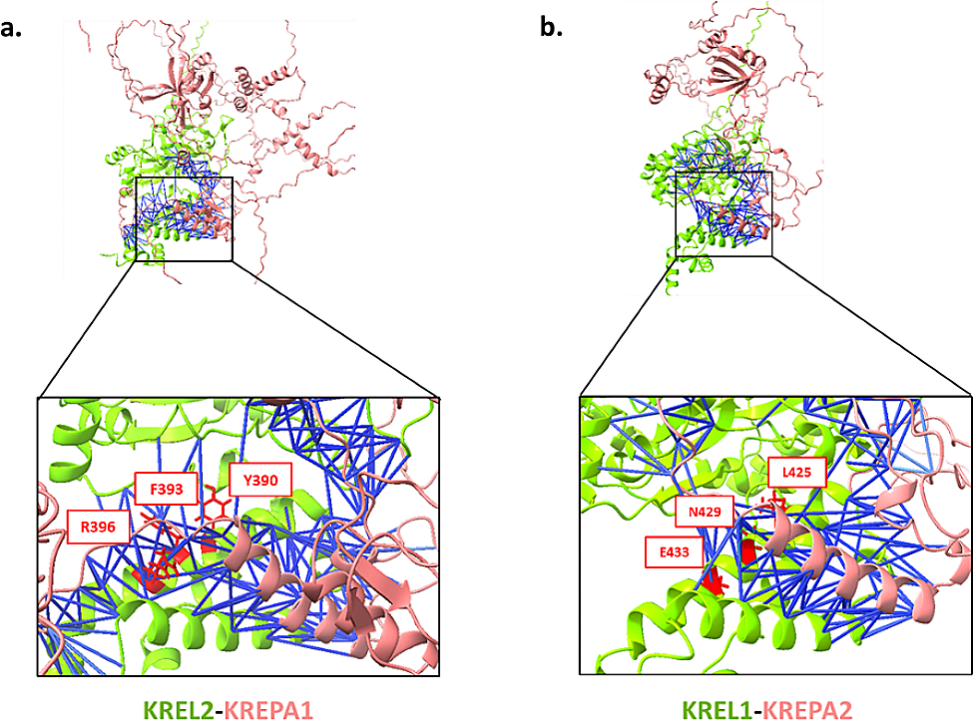
The modeled structure of central editosome ligases interface. (**a**) KREL2-KREPA1 and (**b**) KREL1-A2. Blue lines are the interactions between interface residues of the two interactors (PAE max 5Å, distance max 5Å). Top 3 hotspots of each PPI are labeled and are shown in red with the sidechains visible

Structural analysis of the interfaces of both ligases’ interactions shows a similar pattern in which the ligases primarily interact through their C-Terminal regions with a single alpha helix domain in their interacting partners, KREPA1 and KREPA2. The C-termini of KREL1 and KREL2 consists of four α-helices, with the highest-scoring interface residues located within two corresponding specific helices: aa 392–409 and aa 421–454 in KREL1 as well as aa 353–371 and 381–410, in KREL2 (Fig. 1). These findings are consistent with Moses et al.’s recent research on KREL1-KREPA2, which showed that deletions and mutations in the mentioned helices adversely impact ligation processes [30]. As depicted in Fig. 1, the interface spans both KREL1 and KREL2’s C-termini, where the alpha helices interact most favorably, whereas the remaining interface residues are located within loop regions. Importantly, the interface residues between the helices exhibit higher average centrality and PAE scores, underlining their critical role in the interaction.

##### PPI interfaces of editosome RESC members

###### RESC2-RESC1 interaction

The interface between RESC2 and RESC1 is the most highly ranked among RESC interactions, based on its average network-based MCDA score. RNAi-mediated knockdown of both proteins confirm their essentiality for parasite growth and their critical role RNA editing [42, 43]. Moreover, the RESC2-RESC1 interaction emerges as a central hub in the RESC PPI network and has been validated recently through cryo-EM structural analysis in *T. brucei* [32]. The AlphaFold model created in this study aligns closely with the cryo-EM structure, showing an RMSD of 0.9.

**Fig. 2:**
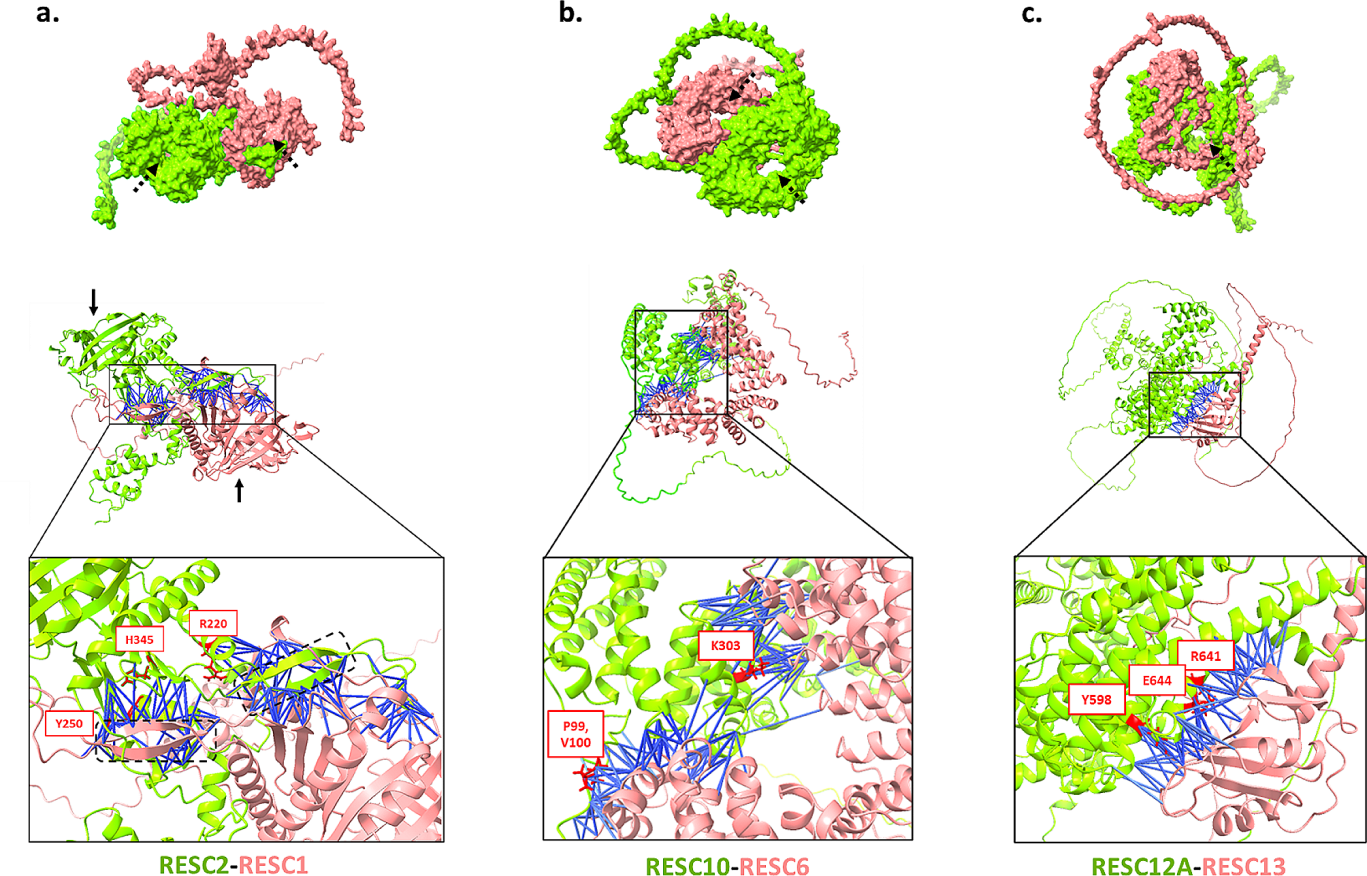
The modeled structures of central RESC PPIs from the editosome of *T. brucei*: (**a**) RESC2-RESC1 PPI, solid black arrows show the beta-barrels of each protein, and the dotted squares show the beta-hairpins; (**b**) RESC12A-RESC13 PPI; (**c**) RESC6-RESC 10 PPI. The upper figure for each PPI is the space filling mode of the PPI structure in which the dotted arrows pinpoint core holes potentially needed for RNA binding. Blue lines are the interactions between interface residues of the two interactors (PAE max 5Å, distance max 5Å). Top 3 hotspots of each PPI are labeled and are shown in red with the sidechains visible

Despite only about 35% sequence similarity, the AlphaFold Multimer model of RESC2-RESC1 reveals a remarkable structural similarity between the two proteins (Fig. 2a). The high-scoring interface primarily involves interactions between a β-hairpin of one protein and a the β-barrel and C-terminal patch of the other. The interface involving RESC2’s hairpin exhibits higher average PAE and RIN centrality values, making it a prime candidate for drug discovery studies.

###### RESC10-RESC6 and RESC12A-RESC13 interactions

The next highest scoring PPIs within the RESC complex are RESC10-RESC6 and RESC12A-RESC13. RESC10 has already been experimentally validated as an essential, yet low abundance, RNA binding protein [44, 45]. RESC10-RESC6’s significance in RNA editing is not yet fully understood. Although in a very recent study Liu et al. which used cryo-electron microscopy and molecular approaches, shed light on this. Even though they did not discuss this interaction, it captured a state of the RESC referred to as the RESC-B particle (PDB ID: 8FNI) [26]. This state showed an interaction between RESC10 and RESC6. A structural alignment of with the AlphaFold-Multimer model showed RMSD of 0.9, suggesting a high degree of structural similarity.

Both RESC10 and RESC6 possess ARM/HEAT repeats in their structural framework. These repeats spiral to form a channel at the core of each protein—a site potentially favorable for RNA binding. Due to inherent flexibility of ARM/HEAT repeats, these proteins can adapt their conformation to align with RNA structures or engage with other proteins. In the PPI model depicted in Fig. 2b, RESC10 engages both ends of RESC6’s spiral curve, effectively obstructing its channel. This complementary interaction implies that in the presence of RESC10 stabilizes RESC6, potentially securing RNA molecule binding. The interface residues at both ends, as well as the predicted hotspots, are supported by good PAE and RIN centrality scores, indicating their importance in this interaction. These critical interface elements are visually represented as blue lines connecting the interacting components in Fig. 2b.

For the RESC12A-RESC13 PPI, existing reports suggest a role in initiating the RNA editing process. These proteins are thought to assist in recruiting other RESC proteins and defining the active editing region. However, detailed studies focusing on this interaction are lacking. The study conducted by Liu et al., separated or excluded RESC12 and RESC12A from the cryo-electron microscopy (cryo-EM) material, thus not providing direct evidence of their interaction with RESC13 [26].

In RESC12A-RESC13 PPI, the N-terminal RNA recognition motif (RRM) of RESC13 mediates its binding to RESC12A (Fig. 2c). While most of the interface residues from RESC12A interact with connecting loops of the RRM domain, the predicted interaction hotspots are primarily located in the defined secondary structures of the RRM, mainly beta-sheets. Interactions with residues in these beta sheets may be especially important for several reasons [46]: (1) they often form strong hydrogen bonds and hydrophobic interactions, contributing to the stability of the PPI, (2) beta sheets tend to be more evolutionarily conserved than loop regions, suggesting the interactions involving these residues are likely essential for the protein’s function. Therefore, even among the predicted hotspots, some might be biologically more relevant which helps in target-based drug design process.

In general, considering the high centrality of RESC12A in the editosome PPI network and its prominent interface ranking with RESC13, further investigation of this interaction could be of significant interest [47, 48]. Additionally, a separate study measuring the stoichiometry (relative abundance) of RESC10 in comparison to other RESC proteins in *T. brucei* has indicated that RESC10 is relatively less abundant, accounting for only approximately 1–2% of the levels of RESC13 and RESC12A [45]. While the low relative abundance of RESC10 compared to other RESC proteins may be indicative of the protein to be present in only a subset of RESC complexes or that it interacts transiently and with RESC complexes [45], it also suggests that RESC10’s lower abundance and transient interactions are due to tight regulation mechanisms make it an interesting target for further studies.

Accordingly, due to the significance of proteomics data in further interpreting the central PPI findings of the editosome, the following section delves into the utilization of this data to enhance the analysis of the results from the previous sections.

### Enhancing editosome PPI investigations with quantitative proteomics analysis

The inclusion of quantitative proteomics data in the current research serves a multiple purpose. Firstly, it can serve as a means of cross-validation for the study’s findings as well as serving as an independent method to find novel central editosome proteins via performing DEA analysis. Moreover, given that one of the primary objectives of the study is to prioritize drug targets, the integration of this data reinforces the fact that the chosen targets are firmly rooted in the disease’s underlying biology. In general, as integrating proteomics data significantly enhances the likelihood of identifying viable and efficacious drug target candidates, it aids in identifying targets that are more likely to translate into effective therapies and ultimately improves the success rate of relevant studies. While acknowledging the benefits of such pipeline, to further enhance the robustness of our findings, we emphasize the necessity of experimental validation. Furthermore, The feasibility of applying a similar pipeline to other pathogens hinges on the accessibility of comparable omics data.

Quantitative proteomics data from [17], is generated from samples of different life stages of *T. brucei* in which long slender bloodstream forms (LS) were differentiated to short stumpy forms (SS) followed by differentiation to insect form trypanosomes (PF) after multiple sampled timepoints in between. Understanding the LS to SS transition is crucial for understanding the lifecycle of *T. brucei*, which has implications for disease progression in the host organism. Furthermore, the proteins that are differentially expressed or interact differently during this transition could be potential targets for therapeutic interventions. Given these factors, the choice to focus on the LS to SS transition offers a well-defined and biologically meaningful context within which to explore the hub proteins and PPIs identified in this study. Therefore, to further investigate the hub proteins and PPIs found in the present study, the LS to SS differentiation was chosen to study DEA as well as GSA.

**Fig. 3:**
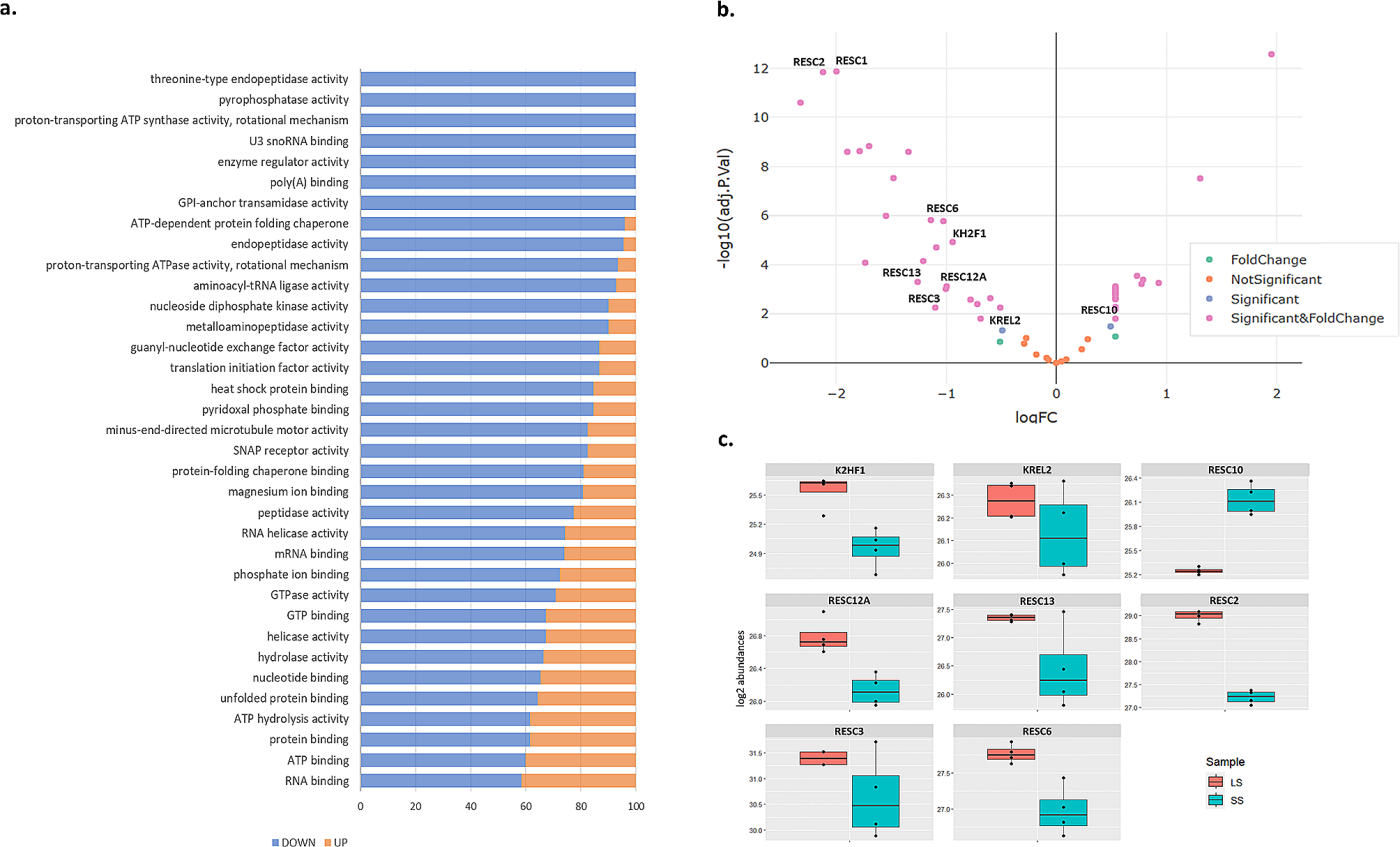
Proteomics data analysis in SS compared to LS form. (**a**) GSA of protein expression Protein sets are defined by GO terms at biological process level. For each GO term showing significant enrichment (in this figure: adjusted p-value < 0.05), the direction of the relative changes in protein levels are shown. (**b**) volcano plot highlighting differentially expressed proteins in (|log2 FC| > 0.5, p-value < 0.05). The detected hubs in this study are labeled on the volcano plot. (**c**) box plots of hub proteins visualizing the protein abundance change

To gain a pervasive view on the differential proteome composition and to put single protein alterations into a larger biological context, gene set analysis (GSA) was performed on all protein abundances from LS and SS samples. To accomplish this, each protein was annotated with Gene Ontology (GO) terms obtained from various databases. Using the created *T. brucei* GO dataset consisting of 35,448 GO terms, GSA showed 35 GO terms to be significantly (adj-*p-*value < 0.05) downregulated in SS form., Most upregulation pattern was observed for members of RNA binding GO terms(Fig. 3a). Given that the transition to the stumpy form marks a change from proliferative to a non-proliferative state, it is reasonable to expect downregulation of different metabolic genes that were more active in the LS form. Importnatly, GSA also identified significant terms related to mitochondrial RNA editing. As illustrated in Fig. 3a, GO terms such as poly (A) binding, RNA helicase activity, mRNA binding and RNA binding include editosome proteins, thereby validating these proteins as promising drug targets due to their critical roles in the parasite’s infective stage.

In addition, DEA performed on the editosome fraction of the proteomics data revealed that all detected hub proteins (see Table 1), except KREL1, showed differential expression during the LS to SS differentiation (Fig. 3b). This suggests that the expression of these central proteins’ expression is significantly upregulated in the proliferative LS, further supporting their potential as promising drug targets.

According to the DEA results, the most significant upregulated editosome proteins ((|log2 FC| > 1), are KREPB4 and KREX2. KREPB4 has been confirmed as an essential protein in both the bloodstream and procyclic forms of T. brucei, whereas KREX2 is not essential in any form of the parasite [49, 50]. These editosome proteins had MCDA scores of approximately 0.55, which did not meet the set threshold for classification as hub proteins in the editosome PPI network. Furthermore, its interactions failed to meet the criteria of being supported with at least one RNase positive assay thus not included in interface analysis in this study.

Figure 3c visualizes the changes in abundance of the top hub editosome proteins in LS and SS forms. All hub RESC proteins, except for RESC10, display significantly reduced abundance in SS form. Previous report has shown that RESC10 interacts with RESC components early in their assembly and then is released. Moreover, the effect of RESC10 on RESC assembly has been reported to be particularly important during transitions between insertion and deletion RECCs [45]. Therefore, its upregulation in the SS form likely reflects the parasite’s adaptive strategy in preparation for its transition to the tsetse fly vector. This facilitates rapid switching between RECC insertion and deletion subcomplexes and thus establishes RESC10’s status as a pivotal member of the editosome complex.

In the next step, after investigating the top ranked hubs via proteomics data, this data was further leveraged to validate the investigated central PPIs (see Table 3) using co-expression analysis. This analysis involves assessing the correlation in expression patterns between the pairs of proteins (PPIs) across different developmental stages of *T. brucei*. Such analysis can help prioritizing hub PPIs based on experimental data; a positive correlation across different timepoints can verify that the PPI is both stable and crucial rather than transient.

**Fig. 4:**
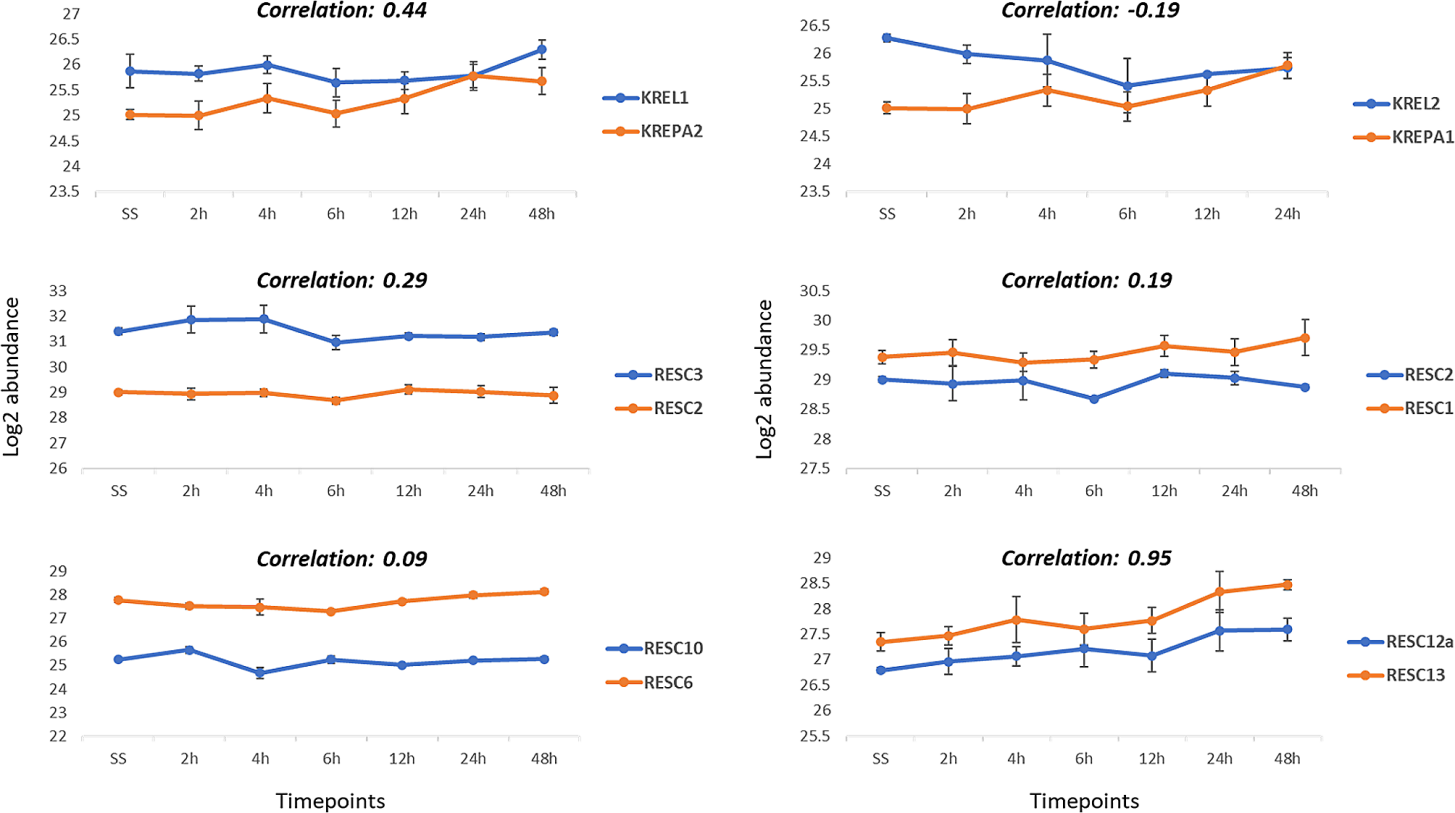
Correlation assessment of the top editosome PPIs using proteomics data from different time points of developmental stage in T. brucei starting from SS as starting point. Correlation coefficient is labeled above the temporal abundance of each graph

As illustrated in Fig. 4, most hub PPIs exhibited a positive correlation between their respective binding interactors, suggesting a potential coordination in the expression or regulation of these protein pairs under various conditions. However, the PPI between KREL2 and KREPA1 exhibited an overall negative correlation yet showed a significant positive correlation during the later developmental time points, specifically, from 6 to 48 h. This temporal correlation pattern is particularly intriguing as it coincides with the parasite’s transition towards fine-tuning its differentiation into the procyclic form.

In contrast, the paralogous PPI, KREL1-KREPA2, demonstrated a very high correlation during the early phase, specifically at the SS-6 h timepoint. This early correlation suggests that KREL1 and KREPA2 may play crucial roles in the initial stages of parasite development. Given that KREL1-KREPA2 and KREL2-KREPA2 are known to be deletion and insertion RECC subcomplexes, respectively [41], our finding highlights the predominance of deletion processes in the SS form, while insertion processes are likely to be more dominant during the parasite’s differentiation into the procyclic form which is the proliferative state of the parasite in the fly vector. As insertion editing is generally more prevalent, that could be indicative of a broader biological strategy for adaptability and complexity, particularly useful during the life stage where the parasite is actively growing and dividing. Furthermore, RESC12A-RESC13 have shown to be significantly correlated throughout the developmental stages of *T. brucei*, making them an interesting case for further analysis specially for drug target discovery procedures.

Overall, these findings highlight the importance of context-dependent analysis when studying protein interactions within the complex biological systems of parasitic organisms. Further investigation into the functional implications of these correlation patterns on the hub editosome PPIs may provide valuable insights into the regulatory mechanisms governing the parasite’s developmental transitions.

## Conclusion

The emergence of resistance to treatments for human African trypanocides emphasizes the pressing need for new therapeutics. Often, resistance emerges due to mutations or alterations in parasite’s receptors or key proteins, making the need for novel drug targets against T. brucei more urgent. However, the lack of functional knowledge about many genes and proteins in the parasite has significantly hindered this endeavor.

Unique to kinetoplastids, the RNA editing process orchestrated by the editosome protein supercomplex provides an excellent opportunity for target-oriented drug discovery. This mechanism is indispensable for the parasite’s survival and adaptability, making it a prime candidate for pharmacological intervention. PPIs lie at the core of biological processes, including RNA editing. Comprehensive structural analysis of these PPIs not only sheds light on our understanding of the underlying biology but also guides the development of drugs capable of modulating these interactions, potentially leading to novel treatments.

Hence, this study employed a multi-layer approach, including structural, network, and omics-based methods, to focus on the most promising drug targets within the central editosome complex in *T. brucei*. By conducting comprehensive analyses and the implementation of state-of-the-art tools like AlphaFold-Multimer, we identified key editosome hub proteins and detailed their pivotal interactions. Our network-centric analysis allows for the prioritization of specific PPIs, with interactions like RESC2-RESC1, RESC12A-RESC13, RESC10-RESC6 and KREL2-KREPA1, standing out for further exploration. Calculating hotspot residues for each interface provides valuable insights for drug candidate selection and functional studies, offering new ways for targeting that might mitigate the likelihood of resistance development.

In conclusion, this study highlights the potential of exploring editosome protein interactions to uncover vital pathways for therapeutic intervention against *T. brucei*. The findings not only underscore the importance of these interactions but also offer a streamlined workflow of integrating multiple analytical approaches for drug target identification, applicable to a wide range of pathogens, pending further experimental validation.

### Electronic supplementary material

Below is the link to the electronic supplementary material.

Additional File 1: Editosome PPI network table of *T. brucei*.

Additional File 2: Centrality analysis results of PPI network.

Additional File 3: RIN-based centrality analysis of hub PPIs integrated with PAE values for interface residue ranking.

## Data Availability

All data generated or analysed during this study are included in this published article [and its supplementary information files].
